# Feasibility of a multi-modal exercise program on cognition in older adults with Type 2 diabetes – a pilot randomised controlled trial

**DOI:** 10.1186/s12877-017-0635-9

**Published:** 2017-10-16

**Authors:** M. L. Callisaya, R. M. Daly, J. E. Sharman, D. Bruce, T. M. E. Davis, T. Greenaway, M. Nolan, R. Beare, M. G. Schultz, T. Phan, L. C. Blizzard, V. K. Srikanth

**Affiliations:** 1 Menzies Institute for Medical Research Tasmania, University of Tasmania, 17 Liverpool Street, Hobart, Tasmania Australia; 2 Stroke and Aging Research Group, Department of Medicine, Southern Clinical School, Monash University, Clayton, Victoria Australia; 3 Institute for Physical Activity and Nutrition (IPAN), School of Exercise and Nutrition Sciences, Faculty of Health, Deakin University, Melbourne, Victoria Australia; 4 Medical School, Faculty of Health and Medical Sciences, University of Western Australia Fremantle Hospital, Fremantle, Western Australia Australia; 5 Murdoch Children’s Research Institute, Royal Children’s Hospital, Melbourne, Australia; 6 Peninsula Clinical School, Central Clinical School, Monash University, Melbourne, Victoria Australia; 7 Department of Medicine, Frankston Hospital, Peninsula Health, Melbourne, Victoria Australia

**Keywords:** Dementia, Cognition, Type 2 diabetes, Exercise, Intervention, MRI brain

## Abstract

**Background:**

Type 2 Diabetes (T2D) is associated with increased risk of dementia. We aimed to determine the feasibility of a randomised controlled trial (RCT) examining the efficacy of exercise on cognition and brain structure in people with T2D.

**Methods:**

A 6-month pilot parallel RCT of a progressive aerobic- and resistance-training program versus a gentle movement control group in people with T2D aged 50–75 years (*n* = 50) at the University of Tasmania, Australia. Assessors were blinded to group allocation. Brain volume (total, white matter, hippocampus), cortical thickness and white matter microstructure (fractional anisotrophy and mean diffusivity) were measured using magnetic resonance imaging, and cognition using a battery of neuropsychological tests. Study design was assessed by any changes (during the pilot or recommended) to the protocol, recruitment by numbers screened and time to enrol 50 participants; randomisation by similarity of characteristics in groups at baseline, adherence by exercise class attendance; safety by number and description of adverse events and retention by numbers withdrawn.

**Results:**

The mean age of participants was 66.2 (SD 4.9) years and 48% were women. There were no changes to the design during the study. A total of 114 people were screened for eligibility, with 50 participants with T2D enrolled over 8 months. Forty-seven participants (94%) completed the study (23 of 24 controls; 24 of 26 in the intervention group). Baseline characteristics were reasonably balanced between groups. Exercise class attendance was 79% for the intervention and 75% for the control group. There were 6 serious adverse events assessed as not or unlikely to be due to the intervention. Effect sizes for each outcome variable are provided.

**Conclusion:**

This study supports the feasibility of a large scale RCT to test the benefits of multi-modal exercise to prevent cognitive decline in people with T2D. Design changes to the future trial are provided.

**Trial registration:**

ANZCTR 12614000222640; Registered 3/3/2014; First participant enrolled 26/6/2014, study screening commenced 1/9/2014; Australian and New Zealand Clinical Trial Registry.

## Background

Worldwide there are over 35 million people living with dementia, with numbers projected to reach close to 115 million by 2050 [1]. Dementia is the greatest cause of disability and associated with significant decline in quality of life. Dementia was recently highlighted as a major societal issue due to the absence of effective disease-modifying medications [2]. Strategies for prevention in people at high risk are therefore important in reducing the current and future burden of dementia on the individual and the health-care system.

Type 2 Diabetes (T2D) is regarded as one of the most important modifiable risk factors for dementia [1]. T2D is associated with a nearly two-fold increase in the risk of incident dementia [3] and with accelerated rates of cognitive decline [4]. The predominate cognitive domains affected include executive function and processing speed, and to a lesser degree verbal and visual memory [5]. The pathways leading to cognitive decline in T2D appear to be mixed with both neurodegeneration [6, 7] and cerebrovascular disease [7, 8] playing roles. Given the presence of multiple risk factors and pathophysiological mechanisms (dysglycaemia, insulin resistance, obesity, hypertension, physical inactivity) [9, 10] contributing to T2D related dementia, therapeutic approaches that can address multiple risk factors simultaneously are likely to provide the greatest benefits.

Structured exercise training for at least 3 to 6 months has been demonstrated to improve glycaemic, metabolic, inflammatory and vascular profiles in people with T2D [11]. Exercise for animals has also been shown to improve angiogenesis, synaptogenesis and neurogenesis [12]. Although there is persuasive evidence from observational studies regarding the benefits of exercise for cognitive health [13], results from human randomised controlled trials (RCT) have been inconsistent in older people [14–19]. A recent meta-analysis of studies of exercise intervention trials in older adults suggests benefits for cognition, for both aerobic and resistance training [20]. However, a Cochrane review and a recent RCT found no benefit for exercise in cognitively healthy older people [14, 15]. Those with T2D represent a distinct high-risk group who have a cluster of biological risk factors for cognitive decline. They may therefore respond favourably to risk factor optimization via a multi-modal exercise program, but the relevant data are limited. A small study of people with pre-diabetes or recently diagnosed diabetes (*n* = 28) found that 6 months of aerobic training improved executive function [21]. In an exploratory post-hoc analysis from the Lifestyle interventions and Independence for Elders (LIFE) RCT (*n* = 415), a 24 month exercise intervention resulted in better scores in global cognitive function and delayed recall only in people with T2D [22]. To date, there has been no adequately powered trial that has demonstrated the efficacy of exercise on cognitive function and other measures of brain health in people with T2D.

Prior to embarking on such a large scale trial, we conducted a pilot RCT in people with T2D to: 1) determine the feasibility (design, recruitment, screening, randomisation, adherence, safety and retention) of a 6-month multi-modal exercise training program compared with control to preserve or improve cognition and brain structure, and 2) provide an estimate of the effects of the intervention on outcomes and MRI brain structure.

## Methods

### Study design

Cognition and Diabetes in Older Tasmanians – A pilot RCT of exercise (CDOT-X) was a 6-month single blind parallel RCT incorporating a supervised and progressive multi-modal exercise program with blinded outcome assessments. Participants with previously diagnosed T2D were randomised to either: 1) a multi-modal aerobic and resistance training (ART) program, or 2) a stretching/gentle movement control group. Participants were randomised by a central automated allocation procedure (parallel groups stratified within two age strata of 50–64 and 65–75 years) after baseline assessment, based on computer-generated random numbers ensuring allocation concealment. Assessment staff were blinded to randomisation group. Participants were informed that the study was designed to examine the difference between two different types of exercise, but were unaware of the nature of the alternative exercise program. All assessments and exercise classes (the two study groups trained at separate times) occurred at the Menzies Institute for Medical Research, University of Tasmania. Brain MRI scans were performed at the Royal Hobart Hospital, Tasmania, Australia. The trial was registered with the Australian New Zealand Clinical Trials Registry: ACTRN: 12,614,000,222,640 and was performed according to the CONSORT 2010 extension to randomised pilot and feasibility trials [23]. Ethics approval was from the Human Ethics Committee Tasmania Network (H0013664) and written informed consent was obtained from all participants.

### Participants and eligibility criteria

Recruitment strategies included contacting participants from a previous longitudinal study of brain health in T2D (Cognition and Diabetes in Older Tasmanians (CDOT) [7], advertising at the Diabetes Clinics at the Royal Hobart Hospital, and by using Australian National Diabetes Services Scheme listings, general advertising, and social media . Eligibility was initially determined by a telephone screening interview (for the inclusion and exclusion criteria listed below). This was followed on a separate day by a medical assessment (including medical history, medication use and an ECG) by a doctor and an exercise stress test at the Menzies Institute for Medical Research. During the test VO2max was collected using the Bruce protocol and an exercise ECG and echocardiogram were performed. If the doctor had any concerns about the participants results they were referred for a cardiology review. Participants who passed these assessments were asked to return on a separate day for the clinic assessment and MRI scan.

#### Inclusion criteria

1) established T2D diagnosed by fasting blood glucose ≥7 mmol/L or 2 h post-prandial glucose ≥11.1 mmol/L or HbA_1C_ ≥ 6.5% according to current American Diabetes Association Guidelines [24]; 2) age 50–75 years; 3) willingness and ability to participate in a structured exercise program for 6-months, and 4) ability to participate in all cognitive testing.

#### Exclusion criteria

1) Severe orthopaedic or respiratory conditions that would preclude participation in an exercise program, or those with absolute contraindications to exercise according to American College of Sports Medicine guidelines [25]; 2) severe cardiovascular disease detected by an exercise stress test as per Australian guidelines [26]; 3) dementia (Telephone Interview for Cognitive Status TICS–Modified score < 27/50); 4) known central nervous system disorders that may have important effects on cognitive function (e.g. intracranial tumour, multiple sclerosis, Parkinson’s disease); 5) contra-indication to MRI; 6) existing participation in structured exercise greater than the equivalent of 30 min per week at moderate intensity.

### Aerobic and resistance training intervention

Participants in the intervention group were asked to undertake a 6-month multi-modal exercise program incorporating aerobic and progressive resistance training (PRT) as this has been found to have the strongest benefit on both cognition [18] and T2D markers such as glucose control [27], as well as potentially benefiting brain health through different signaling pathways [28]. It was implemented by accredited exercise physiologists (AEP) or physiotherapists, who supervised small groups (6–8 people) undertaking two one-hours supervised sessions per week on non-consecutive days, supplemented by a further one-hour per week unsupervised session at the participant’s home or in the community with prescribed aerobic exercise. Intensity of training relied on the Borg Scale rating of perceived exertion (RPE; range 6 to 20) [25]. Each supervised session consisted of approximately 30 min of moderate- to high-intensity PRT (3 sets of 8–12 reps at 14–17 ‘hard to very hard’ on the 20 point RPE scale) of the upper and lower extremity exercises using body weight, machine or free weights [29]. An aerobic component of approximately 30 min consisted of stationary cycling, cross trainer, rower or treadmill walking starting at a low-moderate intensity (RPE 12–13) and progressively building to a moderate-vigorous intensity (RPE 14–16) [26]. The program was individually tailored to each participants ability and progressive intensity was applied with increasing fitness to ensure safety recommendations were met [26]. The 6-month program was divided into six 4 weekly mesocycles and a final 2 week mesocycle of progressing higher intensity. Within the 4 week cycle the final week was at a slightly lower intensity (regeneration phase), before increasing intensity again in the first week of the following mesocycle.

### Control intervention

Participants randomised to the control arm received a 6-month light intensity upper and lower limb stretching and gentle movement program, which was also implemented by AEPs and physiotherapists at a distinct time to the intervention group. Participants attended two one-hour supervised sessions per week, and carried out one unsupervised session at home. These activities were designed to provide the same amount of participative involvement and socialization as the intervention group.

Both groups were provided with a participant manual outlining the group specific exercise program as well as motivational tips and safety advice (e.g. foot inspection, measurement of blood glucose before and after exercise).

### Feasibility measures

#### Design

Ability and time required of study staff to co-ordinate the recruitment, screening and clinic tasks; co-ordination of exercise and control group classes; ability of participants to attend classes; any changes to the study protocol.

#### Recruitment and screening

Time and number of people screened to enrol 50 participants.

#### Randomisation

Balance of characteristics in each group.

#### Adherence

Adherence in both groups assessed by attendance at the supervised sessions. Percentages of classes attended/allocated in each group. Home exercise training was assessed by a participant exercise diary.

#### Safety

The number and description of both serious (any admission to the department of emergency medicine or hospitalization, life threatening event or results in persistent or significant disability/incapacity or death) and other adverse events by group. Serious adverse events (including falls, injuries, hypoglycaemic events and foot ulcers) were adjudicated by a Data Safety Monitor (an independent academic General Practitioner) as not related to the study, probably not related, unlikely but possibly related or probably related to the study.

#### Retention

Number of participants that withdrew by group.

### Outcome measures

Measurements were administered at baseline and 6-months at the Menzies Institute for Medical Research, apart from MRI scans. Clinics lasted approximately 3 h and included breakfast. MRI scans occurred on a separate day at the Royal Hobart Hospital which is located across the road from the Menzies Institute for Medical Research and took approximately 1 h. At the 6 month visit participants were asked not to tell assessment staff which group they attended. All data (except MRIs) were collected on teleform questionnaires, which were scanned and transferred to a secure access database.

#### Brain MRI

The following acquisition protocols were used in a 3.0 T GE Signa HDxt scanner: High-resolution 3-dimensional T1 weighted spoiled gradient recalled (SPGR) (TR = 6.732 ms, TE = 2.816 ms, flip angle =12°, FOV = 225 mm, voxel size 1x1x1mm; fluid attenuated inversion recovery (FLAIR, TR = 10,000 ms, TE = 121 ms, FOV = 220 mm, flip angle = 90°, voxel size 0.4 × 0.4 and 4 mm); DTI using High Angular Resolution Diffusion Imaging (HARDI) sequence with 64 gradient directions (b = 2000s/mm^2,^ TR = 7200 ms, TE = 105 ms, FOV = 240 mm, voxel size (2.0.954 × 0.952.4 × 6.53 mm3) and 1 b = 0 s/mm^2^ reference image. MRI pre-processing and analyses were performed in the image analysis lab at Monash University using well-established methods blinded to age, sex and outcome measures. Brain structural outcome measures included total brain, white matter and hippocampal volume and cortical thickness. The Freesurfer 5.3 longitudinal pipeline [30] was used to estimate cortical thickness and brain volume. Hippocampal volumes were estimated using FSL FIRST [31] with the following additions to improve reliability on older participants. The T1 weighted scans were nonlinearly transformed to MNI space using the transformations estimated by the SPM12 unified segmentation procedure. FIRST was applied to the transformed image and the resulting segmentation transformed back to native space for volume estimation. The FSL TBSS [32] preprocessing pipeline was used to compute a white matter skeleton of the whole brain. Mean white matter microstructural measures, fractional anisotropy (FA) and mean diffusivity (MD), were computed for the skeleton. Cerebrovascular white matter hyperintensities were delineated as previously described [33], with the statistical classifier stage replaced by a manual selection. Lesion masks were used to correct Freesurfer errors caused by hypointensities on T1-weighted scans.

### Cognitive function

The following tests were administered: 1) The Victoria Stroop test (interference score C-D) [34]; 2) The Trail Making Test (shifting score B-A); 3) The Digit Symbol Coding Test (DSC) [35]; The digit span subtest of the Wechsler Adult Intelligence Scale – Third Edition (WAIS-III) [35]; Controlled Oral Word Association Test (COWAT) [36]; The three part Hopkins Verbal Learning Test – Revised (HVLT) [36] and the Rey Complex Figure copy and delay [36]. These tests were chosen as they are sensitive to the effects of T2D [5], and mild cognitive changes that affect daily functioning, and have previously been used in intervention studies in T2D to preserve cognition [37]. In post-hoc analysis we used the cognitive measures that showed improvement in the ART group compared to the control group to calculate a global composite score similar to prior studies [38, 39].

### Other measures

The following measures were also assessed: 1) *Brachial and central blood pressure* using the IEM Mobil-O-Graph with 8 automated readings taken seated every 2 min and then averaged; 2) *Fasting blood tests*: blood glucose, insulin, HbA_1C_ (baseline only) using standard techniques; 3) *Physical fitness*: VO_2_max obtained using the Bruce treadmill protocol measured on a separate day to the other clinic assessments; 4) *Knee extension strength* (kg) using a spring gauge from the Short Physiological Performance battery [40] and *grip strength* using a Jamar digital hand dynamometer (kg); 5) *Anthropometrics:* Height and weight to calculate BMI and waist circumference using standard measures; and 6) *Health and lifestyle*: A standardised questionnaire was used to collect data on health, medical history and medication use.

### Statistical analysis

A consort diagram was used to summarise screening, recruitment and study retention. Descriptive statistics were used to describe the characteristics of the sample by group (means [SD], percentages and frequencies) as well as exercise adherence and adverse events. Cognitive and brain measure at baseline and follow-up were presented as means and SE and change over time as means and 95%CI by group. Random intercept linear mixed models with maximum likelihood estimation adjusted for age, sex and education (and intracranial volume for MRI measures) were performed to determine if there were any differences in the slope between the groups for the change over time. Correlations coefficients were used to examine associations between change in mechanistic variables (VO_2max,_ knee extension strength, waist circumference, central and brachial blood pressure, and fasting glucose and insulin levels) and change in a cognitive global score. As this is a pilot study we have not provided *P*-values or controlled for Type 1 errors, but have included mean changes with 95% confidence intervals (CI) for the within group changes and the net between group differences for the change over time to provide an indication of the magnitude and direction of any effect [41].

## Results

### Design

**Fig. 1:**
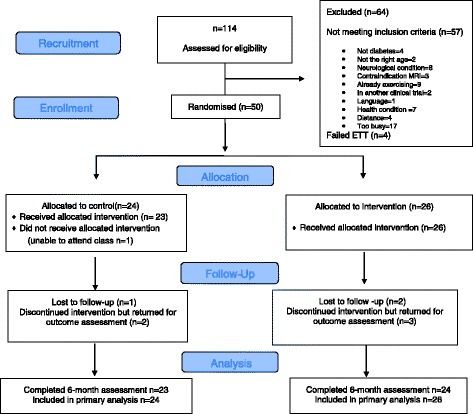
Consort flow diagram

Coordination of recruitment, screening, clinic assessments (3–3.5 h per person) and the interventions were performed without any changes to the protocol during the pilot. Offering classes at multiple times worked well with only one participant unable to attend the two classes per week (see CONSORT diagram Fig. 1). The class size of 6–8 participants in the intervention group (1 h) and 8–10 participants in the control group (1 h) was deemed essential to enable adequate supervision to ensure safety and protocol adherence. Trainers noted that an extra 15–20 min for new participant orientation and for completing exercise charts would be useful. Onsite parking was also considered important by participants.

### Recruitment, screening and randomisation

One-hundred and fourteen participants were screened for eligibility of whom 50 (mean [SD] age 66.2 [4.9] years; 48% women) were randomised and assigned to the intervention or control group over an 8-month period (September 2014 to May 2015). Reasons for exclusion are shown in Fig. 1 and group characteristics are summarized in Table 1.

**Table 1:** Baseline characteristics of the total sample, ART and control group

Characteristics, mean, SD unless indicated	Total (*n* = 50)		ART intervention (*n* = 26)		Control (*n* = 24)	
Age, years	66.2	4.91	65.3	5.0	67.1	4.8
Female, n (%)	24	(48.0)	15	(57.7)	9	(37.5)
Body mass index, kg/m^2^	30.9	4.87	31.1	5.2	30.7	4.7
Years of education, n (%)	12.6	(3.4)	13.3	(3.6)	11.8	(3.1)
HbA1c, %	6.8	1.1	6.8	1.2	6.8	1.0
Fasting glucose, mmol/L	8.3	2.6	8.5	3.0	8.1	2.1
Fasting insulin, mU/L	19.1	20.6	17.8	19.5	20.6	22.2
Systolic blood pressure, mmHg	117.6	12.2	117.2	10.1	118.0	14.5
Diastolic blood pressure, mmHg	77.6	10.4	77.9	8.0	77.3	12.8
VO_2max_, L/min	21.6	4.8	22.0	5.1	21.1	4.5
Self-reported medical history						
Hypertension, n (%)^a^	44	(88.0)	21	(80.8)	23	(95.8)
Insulin use, n (%)	10	(20.0)	6	(23.1)	4	(16.7)
High Cholesterol, n (%)	42	(84.0)	21	(80.8)	21	(87.5)
Myocardial infarction, n (%)*	2	(4.1)	0	(0.0)	2	(8.3)
Years since Type 2 Diabetes diagnosis	12.6	7.1	11.8	7.6	13.3	6.5

*ART* aerobic and resistance training, *HbA1c* glycated hemoglobin, *SPPB* short physical performance battery

^*^one participant had missing data

^a^Hypertension: self-reported hypertension or SBPmmHg > 140

***Retention:*** Of the 50 participants, 47 (94%) remained in the study at the 6-month follow-up. Reasons for withdrawal (ART *n* = 2; control *n* = 1) were unexpected travel interstate (*n* = 1), carer for family member (*n* = 1) or inability to contact (*n* = 1). Of the cognitive assessments one person was colour blind and could not complete the Stroop test. Three people had missing components of the HVLT (delay and recognition [*n* = 1]; recognition [*n* = 2]). Of the 47 participants who returned at 6 months, 1 further participant was unable to complete the MRI scan due to new claustrophobia. ***Adherence:*** Exercise class attendance for the ART group was 79% and for the controls 75%. Seventy five percent of participants adhered to the 60 min of home exercise.

### Brain MRI

Table 2 provides the unadjusted and adjusted means for brain MRI measures at baseline and 6 months, the mean change over time in each group, and net between group differences for the change over time (with 95% CI). Compared with the controls, the ART group showed improvements in white matter integrity (FA), hippocampal and total brain volumes, and less decline in white matter volume over the 6-month period.

**Table 2:** Mean baseline and 6-month scores for cognitive function and brain MRI and the mean within group changes and net between groups differences for the change over time

Outcome	ART group						Control Group						Between-group difference^a^	
	Baseline		6-months		Change		Baseline		6-months		Change			
	Mean (SE)		Mean (SE)		Mean (95% CI)		Mean (SE)		Mean (SE)		Mean (95%CI)		Mean (95% CI)	
Cognitive global score	0.06	0.11	0.20	0.11	0.14	0.02,0.27	−0.06	0.12	−0.08	0.12	−0.01	−0.14,0.12	0.16	−0.02, 0.34
RCF copy	27.6	0.9	27.4	0.9	−0.2	−1.8, 1.5	26.2	0.9	25.9	0.9	−0.3	−2.03,1.4	0.2	−2.1, 2.6
RCF delay	14.8	1.0	16.0	1.0	1.3	−0.2, 2.8	13.9	1.0	15.5	1.00	1.6	0.03, 3.1	−0.3	−2.5, 1.8
Stroop C-D	16.7	1.8	12.9	1.9	−3.8	−7.1, −0.5	20.1	1.9	18.3	1.9	−1.8	−5.1, 1.6	−2.1	−6.7, 2.6
Trails B-A	40.2	4.5	31.5	4.7	−8.7	−17.9,0.5	38.2	4.7	33.8	4.8	−4.3	−13.8,5.1	−4.4	−17.7, 9.0
DSC	58.6	2.6	60.8	2.6	2.2	0.2, 4.2	56.1	2.7	56.1	2.7	0.0	−2.1,2.1	2.3	−0.6, 5.2
Digit span	19.2	0.7	18.2	0.7	−1.0	−1.9,-0.1	19.9	0.7	20.6	0.7	0.7	−0.2,1.6	−1.7	−3.0, −0.5
Hopkins I	26.1	0.9	26.8	0.9	0.7	−0.9,2.2	27.0	0.9	27.3	1.0	0.3	0.-1.3,1.9	0.4	−1.9, 2.7
Hopkins D	9.4	0.4	9.5	0.5	0.1	−0.7,0.9	9.0	0.5	9.2	0.5	0.2	−0.6, 1.0	−0.1	−1.2, 1.0
Hopkins R	10.7	0.2	10.5	0.2	−0.2	−0.7,0.4	10.7	0.2	10.1	0.2	−0.6	−1.1,-0.0	0.5	−0.3, 1.2
COWAT word	40.7	2.5	43.7	2.5	3.1	0.4,5.7	38.3	2.6	39.7	2.6	1.4	−1.3,4.1	1.7	−2.0, 5.4
COWAT Category	21.0	0.9	20.1	0.9	−0.9	−2.6,0.8	18.7	1.0	19.3	1.0	0.6	−1.1,2.4	−1.5	−3.9, 1.0
MRI brain														
Total brain	1128.5	28.6	1130.1	28.6	1.6	−2.3,5.5	1176.14	28.55	1175.7	28.6	−0.5	−4.4,3.4	1.9	−3.7,7.4
White matter	742.2	34.7	741.1	34.7	−1.1	−9.9,7.7	814.46	34.66	808.7	34.7	−5.8	−14.6,3.1	4.3	−8.4,17.0
C-thickness	3.8	0.1	3.8	0.1	0.0	−0.1,1.1	3.43	0.13	3.5	0.1	0.1	−0.0,0.2	−0.0	−0.2,0.1
Hippocampal	6.9	0.1	7.0	0.1	0.10	−0.01,0.20	7.2	0.14	7.1	0.1	−0.02	−0.12,0.08	0.13	−0.03, 0.29
White FA	0.408	0.004	0.412	0.004	0.002	−0.002,0.006	0.406	0.004	0.399	.005	−0.007	−0.012,-0.003	0.009	0.003, 0.016
White MD	0.001	0.000	0.001	0.000	−0.000	−0.000,0.000	0.001	0.000	0.001	0.000	−0.000	−0.000,0.000	−0.000	0.000,0.000

*RCF* Rey Complex Figure, *DSC* digit symbol coding, Hopkins (I-immediate, *D* delay, *R* recall), *COWAT* Controlled Oral Word Association Test, *C-thickness* cortical thickness, *FA* fractional anisotrophy, *MD* mean diffusion, Stroop and Trails:higher scores poorer performance

^a^adjusted for age, sex and education (brain measures also adjusted for intracranial volume)

### Cognition

Unadjusted and adjusted means for cognitive measures by group at baseline and 6 months, and the mean change and net between group differences (with 95% CI) are also presented in Table 2. In post-hoc analyses we also created a global cognitive score by forming a composite of these tests calculated as z scores standardized to the baseline mean and SD [4, 14] (Table 2). Compared with controls, the ART group showed improvements in the global cognitive score, Stroop C-D, Trails A-B, DSC, Hopkins intermediate and recognition scores, COWAT-word and Rey Complex Copy tests. Table 3 shows the correlations between change in the mechanistic variables and change in the cognitive composite scores by group. The strongest association was seen between an increase in fitness (VO_2max_) and increase in global cognition (*r* = 0.42), with a weaker association for reduction in central systolic blood pressure (*r* = −0.15). An association between global cognition and fasting blood glucose (*r* = 0.13) was in the opposite direction than expected.

**Table 3:** Pearson’s correlations between change in mechanistic variables and change in global cognitive function in all participants combined

	Waist circumference	VO_2max_	Brachial SBP	Brachial DBP	Central SBP	Central DBP	Knee strength	Fasting glucose	Fasting insulin	Grip strength
Global cognitive score	0.08	0.45	−0.08	−0.10	−0.15	−0.05	0.03	0.13	0.01	0.11

*SBP* systolic blood pressure, *DBP* diastolic blood pressure

### Safety: adverse events

There were two serious adverse events in the intervention group and four in the control (Table 4). All serious adverse events were ruled by the Data Safety Monitor as unlikely or not due to the intervention. One participant suffered a myocardial infarction 1 week after finishing the ART program and this was ruled unlikely but possibly due to the intervention. There were nine other adverse events recorded including two falls in the control group (in the same participant; one fall in the class); one person with symptomatic postural drop during the exercise tolerance test and six (5 ART and 1 control group) with musculoskeletal complaints of which all resolved within a few weeks or specific exercises associated with pain were modified.

**Table 4:** Participants reporting adverse events

	Total	Intervention	Control
Serious adverse events	6	2	4
Cardiac problem^a^	2	1	1
High blood pressure^a^	1	0	1
Joint surgery^a^	1	1	0
Gall bladder^a^	1	0	1
Allergy^b^	1	0	1
Important adverse events	9	5	4
Fall	2	0	2^c^
Musculoskeletal pain	6	5	1
Symptomatic postural drop during ETT	1	0	1

*ETT* exercise tolerance test

^a^Required hospitalization

^b^required Emergency Department admission

^c^Both in the same participant

## Discussion

The present pilot study has demonstrated feasibility (in study design, recruitment, screening, randomisation, adherence, safety reporting and retention, and effect size estimates) for a subsequent definitive RCT to test whether a multi-modal exercise program incorporating aerobic plus resistance training can provide benefits to cognition and brain structure in people with T2D. Recruitment procedures resulted in 114 people fulfilling the inclusion and exclusion criteria volunteering over 8 months (approximately 14 participants per month). A telephone screening procedure was successful in determining eligibility and availability including identifying those who were too busy, lived too far away or who were unable to participate for other health reasons. The face-to-face medical assessment and exercise stress test were useful in excluding a further seven people due to high cardiovascular risk. The rate of recruitment was also perceived by trainers as optimal in order to allocate sufficient time for orientating new participants to exercises. Taking into account the relatively small sample size, baseline characteristics appeared balanced between groups except for sex and prior myocardial infarction. It is likely that a larger sample size would further improve the comparability of the intervention and control groups.

The intervention group combined aerobic and resistance training, which is based on superior outcomes previously observed with cognition [18] and T2D markers such as blood glucose control [27], as well as potentially benefiting brain health through different signaling pathways [28]. The control group carried out stretches and gentle movements designed to provide the same amount of socialization as the intervention group. The adherence for the two centre-based sessions was similar (79% in the intervention and 75% for the control group), which suggests that participants accepted the outcome of randomisation. The retention rate (94%) was comparable or better than prior studies described in a meta-analysis [42] with only three people not returning for follow-up. We established the need for small exercise groups to ensure the program was tailored according to each individual, with close supervision by a trainer to enhance motivation, ensure correct exercise technique and appropriate blood glucose monitoring. Six serious adverse events (all in different participants) were deemed not or unlikely due to the intervention. Of other adverse events, there was one fall without injury that occurred in the control group. Five participants also reported musculoskeletal pain in the intervention group and one in the control group, all of which were pre-existing or resolved with expectant management. It is difficult to compare these figures to other trials as a prior report of adverse events in 121 exercise RCT found that in general, adverse events are poorly reported [43]. In order of prevalence, musculoskeletal complaints, falls and cardiovascular events were most common [43]. Older people and those with T2D are more likely to have conditions such as arthritis and cardiovascular disease. In light of this, we took precautions by performing a medical screen and exercise stress test before randomisation, providing participants with a booklet of the training program and exercise precautions, as well as having a 4 week light intensity preparatory training phase at the start of the program, and one easier week in every 4 week cycle. However, a further individualized initial orientation and assessment at the beginning of the program with the trainer may also be worthwhile.

As this was a pilot study, no a priori power estimations were performed, and no tests of efficacy or statistical significance are provided for the between group differences or change over time in brain or cognitive measures as false positives and false negatives are likely. However, 95% confidence interval were provided to give information about the magnitude and direction of an effect [23, 41]. Nevertheless, our between-group difference for the change over time in the global cognitive score was similar to the change (mean decrease 0.18 SD) observed over 5-years in a previous observational study in people with T2D [4]. These data suggest that a global composite score can be a sensitive primary outcome measure in a larger definitive exercise trial, avoiding issues related to multiple primary outcome measures [44]. Of the individual cognitive scores, all but the Rey Complex Figure delay, Digit span, Hopkins Delay and the COWAT Categories tests favored the intervention group. We included structural brain and cognitive measures that have previously been shown to be sensitive to T2D related cognitive impairment [5, 7, 45]. Few prior RCT have included brain MRI as well as measurements to study underlying pathways influenced by exercise. In the few brain imaging studies of people without T2D, as little as 6–12 months of exercise has been shown to lead to measurable increases volume of the hippocampus [46, 47]. Our pilot data are broadly consistent with these findings and suggest that total, white matter and hippocampal volume, as well as white matter integrity might benefit from an ART program. This needs to be confirmed in a larger trial.

Although we foresee no major changes to the protocol in a larger study, there are a number of considerations in the design and conduct of future such trials. Firstly, 12 participants did not complete the VO_2max_ test at follow-up. Although we did not record the reason for this, wearing a mask for this test and exercising to maximum levels may have caused discomfort to some participants. Sub-maximal tests that reliably estimate VO_2max_ may be easier to complete in future trials. Secondly, although there was no indication that assessment staff were aware of group allocation, we did not formally evaluate blinding such as by asking blinded study staff which group that thought participants were in. Thirdly, exercise trainers indicated that heart rate monitoring would assist in assessing intensity of aerobic exercise training over and above the RPE scale. Fourthly, the treatment period was relatively short. The optimum length of the intervention that is required to prevent long-term cognitive decline is unknown. Prior studies have reported benefits of both kinds of exercise (aerobic and resistance) on blood pressure, glycaemic control, insulin sensitivity, and fat mass within 3–6 months [11]. A meta-analysis of exercise for cognitive function in older adults found the largest benefits with 3–12 months following aerobic plus resistance training [18]. However, many of the benefits of exercise may disappear after a program is stopped. Encouragingly, one study reported that a 24-week walking program was efficacious in improving cognition in older people with MCI one-year post intervention [19]. This suggests that a short-term (6-months) program may boost cognitive reserve over the longer term. A larger definitive study based on this pilot is planned to incorporate and evaluate a 12-month phase designed to assist participants incorporate the program into real world home or community exercise programs. Finally it is possible that some medications such as insulin therapy and the number of adverse events may influence results. These should be considered in the statistical plan and power calculations of a future study.

In addition to the above considerations there are other limitations to the design of our study. The large number of cognitive tests may have led to fatigue and a decline in performance. However the order of tests was similar for all participants. The lack of follow-up over longer periods means that we were unable to determine longer term dropout rates, effects on cognition or conversion to dementia. It is possible that some participants in the control group may have started to exercise, but it is unlikely that they took up training at the intensity performed by the intervention group. They also attended the control classes twice a week which would have left less time to take up another new exercise program. It is possible that the control class may also have provided cognitive benefits, reducing the differences between groups. Although the combination of aerobic and resistance training may provide additional benefit, a four armed study (aerobic, resistance, aerobic and resistance, control) would be required to tease out their individual effects. Finally, some participants were from the longitudinal CDOT study which may limit generalizability. However, these people are well characterized and were also recruited from the Australian National Diabetes Service Scheme.

## Conclusion

In conclusion, the present pilot study demonstrates strong feasibility in terms of design, recruitment, screening, adherence, safety and retention to a multi-modal exercise program. A larger definitive trial to determine if exercise can delay or prevent cognitive decline in this high-risk group is now required. A delay in just 1 year of the onset of dementia may result in 9.2 million fewer cases globally [48].
